# Time-dependent effects of storage at –80 °C on the stability of butyrylcholinesterase activity in human serum

**DOI:** 10.1016/j.plabm.2022.e00298

**Published:** 2022-07-19

**Authors:** Chien-Hui Huang, Yi-Ting Chang, Scott Severance, Jui-Ying Feng, Sin-Yu Hou, Ming-Mao Gong, Chi-Ching Hwang, Chia‐Yen Dai, Jeh-Jeng Wang, Tzu-Pin Wang

**Affiliations:** a Department of Medicinal and Applied Chemistry, Kaohsiung Medical University, Kaohsiung, 80708, Taiwan; b Department of Nursing, College of Medicine, National Cheng Kung University, Tainan, 701, Taiwan; c Department of Molecular and Cellular Sciences, Liberty University College of Osteopathic Medicine, Lynchburg, VA, 24515, USA; d Department of Biochemistry, Faculty of Medicine, College of Medicine, Kaohsiung Medical University, Kaohsiung, 80708, Taiwan; e School of Medicine, College of Medicine, Kaohsiung Medical University, Kaohsiung, 80708, Taiwan; f Department of Internal Medicine, Kaohsiung Medical University Hospital, Kaohsiung, Taiwan; g Department of Medical Research, Kaohsiung Medical University Hospital, Kaohsiung Medical University, Kaohsiung, 80708, Taiwan

**Keywords:** Butyrylcholinesterase, Serum, Activity, Temperature, Repeated measures ANOVA, Sidak multiple comparisons test

## Abstract

**Objectives:**

Butyrylcholinesterase (BChE) is an important biomarker in serum, and aberrant BChE activity indicates onset and progression of human diseases. The duration of serum storage at −80 °C may introduce variability into and compromise the reproducibility of BChE activity measurements.

**Design and Methods:**

We collected serum samples from eight healthy volunteers and determined serum BChE activity in these samples using a sensitive fluorescence assay at various time points during a six-month storage period at −80 °C. Changes in averaged BChE activity over storage time were assessed by repeated measures analysis of variance (ANOVA). Sidak multiple comparisons test was also used to perform post-hoc analysis.

**Results:**

Almost all determined BChE activity values lay within the normal physiological range of BChE activity. However, repeated measures ANOVA using mean BChE activity vs. storage time showed that BChE activity values from two time points were significantly different. Analysis by Sidak multiple comparisons test provided no substantial change of BChE activity during the first 90 days of storage, but BChE activity noticeably decreased after 90 days.

**Conclusions:**

Serum samples stored in −80 °C for up to 90 days can be exploited to accurately determine BChE activity.

## Highlights

• Butyrylcholinesterase (BChE) activity was measured by a fluorescence turn-on assay.
• BChE activity in human serum was determined during 180-day storage at −80 °C.
• Gender difference on BChE activity was not observed according to *t*-test.
• Serum BChE activity maintained stability at −80 °C for up to 90 days.
• A clinical criterion on BChE activity measurements is established.

## 1 Introduction

Butyrylcholinesterase (BChE; EC 3.1.1.8) is the major cholinesterase in human serum and is capable of hydrolyzing many different bioactive esters including acetyl(thio)choline, butyryl(thio)choline, esters in plant-based foods, ester-containing drugs, and the ‘hunger hormone’ ghrelin - an esterified neuropeptide [1,2]. The biological role of serum BChE has been elusive and remains under debate. Nevertheless, abnormal levels of BChE activity have been correlated with the development and progression of many human disorders such as acute inflammatory diseases, diabetes, cardiovascular disease, metabolic syndrome, hepatocellular carcinoma, chronic liver diseases, postoperative delirium and complications, and poisoning with organophosphates and metals [[1], [2], [3], [4], [5], [6]]. BChE activity in human blood is, therefore, a useful biochemical marker with many potential clinical applications [2,3,[7], [8], [9]]. Accurately measuring fluctuations of BChE activity in the serum is critical to reliable and timely diagnoses of disease states. In addition, understanding how physical parameters such as temperature and storage time affect the activity of serum BChE is also essential to corrobating reproducibility and accuracy of BChE activity results when samples are measured after prolonged storage. Limited research has focused on deciphering how BChE activity is affected by short-term storages, such as six months, at specified temperatures [[10], [11], [12], [13]]. Past studies relied on insensitive colorimetric methods to determine BChE activity while storing samples in temperatures higher than −80 °C for short periods. There is an urgent need for medical facilities to not only have straightforward and accurate techniques to quantify BChE activity but also have valid criteria of preanalytical conditions for sample storage in order to reproducibly measure BChE activity when determining BChE activity after storage.

The current research responds to the request for sensitive BChE activity assays capable of determining how temperature and duration of storage affect BChE activity and how reliable measurements of BChE activities obtained from stored samples are. We recently synthesized a novel chemical probe, which was exploited to develop a fluorescence turn-on assay capable of accurately quantifying BChE activity in serum while simultaneously demonstrating a discriminative reactivity toward glutathione (GSH) and avoiding GSH interference in the assay [14]. The sensitive fluorescence assay was employed to measure BChE activity in human serum at various time points throughout a six-month storage period at −80 °C. The BChE activity results were analyzed by repeated measures analysis of variance (ANOVA) and Sidak multiple comparisons test, and BChE activity could be reproducibly measured in serum samples stored at −80 °C for up to 90 days.

## 2 Material and methods

### 2.1 Blood collection and biochemical analysis of BChE activity in the blood samples

Clinical research ethics review board at Kaohsiung Medical University (KMU) had reviewed and approved the study. Consent had been obtained from eight healthy volunteers including four males and four females. A trained phlebotomist at KMU Hospital obtained 20 ml of whole blood from each of the volunteers. Blood analytes were separated to provide the corresponding serum samples which were either analyzed immediately or aliquoted and stored at −80 °C for future use. Fresh serum or thawed serum aliquots from the −80 °C freezer were always used to determine BChE activity the same day, and unused serum was properly discarded after analysis.

A sensitive fluorescence turn-on assay [14] was employed to determine BChE activity of fresh serum or serum samples stored in the −80 °C freezer for 1, 2, 4, 6, 13, 90, 135, or 180 days. The quantitative measurements of BChE activity by the fluorescence chemical probe (designated as **11** in Ref. [14])-based assay in a PerkinElmer LS-55 fluorescence spectrometer are briefly described below. The assay began with kinetic analysis of time-dependent fluorescence turn-on of **11** in the presence of equine BChE standards with different activity to acquire the corresponding values of the initial rate (*v*_i_) and to establish the linear regression relationship of BChE activity vs *v*_i_. In each assay, a 1.5 mL solution containing the fluorescence chemical probe **11** (25 μM), BChE (1.82–182.2 U L^−1^), and the substrate *S*-butyrylthiocholine iodide (BTCh, 5 mM) in phosphate buffer (PB, pH = 7.4, 0.1 M) was prepared in a quartz cuvette previously mounted onto the temperature controller of the fluorescence spectrometer. BChE catalysis was carried out at 37 °C for no longer than 90 min; reaction progression was monitored by the fluorescence (FL) intensity at 505 nm as a function of reaction time. The normalized FL intensity data at 505 nm were acquired by subtracting a background FL_505_ intensity of **11** from the original FL_505_ intensity reading. The FL changes in windows of the steady state reactions were used to calculate *v*_i_ of catalysis in the presence of a specific activity of BChE. The resulting linear regression equation was exploited to determine BChE activity in serum samples with which a 15 μL of the fresh or −80°C-stored serum sample was used to substitute for BChE in the 1.5 mL reaction mixtures for the **11**-based assay. Each assay for serum BChE activity was typically performed for 10 min in order to accurately determine the corresponding *v*_i_ and BChE activity. In addition, all measurements of BChE activity were repeated a minimum of three times to acquire the averaged BChE activity values reported as a mean value ± SD.

### 2.2 Statistical analysis

Gender difference on BChE activity at each time point was assessed using independent *t*-test. Repeated measures ANOVA was used to assess changes over time in mean BChE activity values. Sidak multiple comparisons test was used to perform for post-hoc analysis. A *p*-value < 0.05 indicated statistical significance.

## 3 Results

### 3.1 Measurements of BChE activity in serum samples freshly acquired or stored in −80 °C

**Fig. 1:**
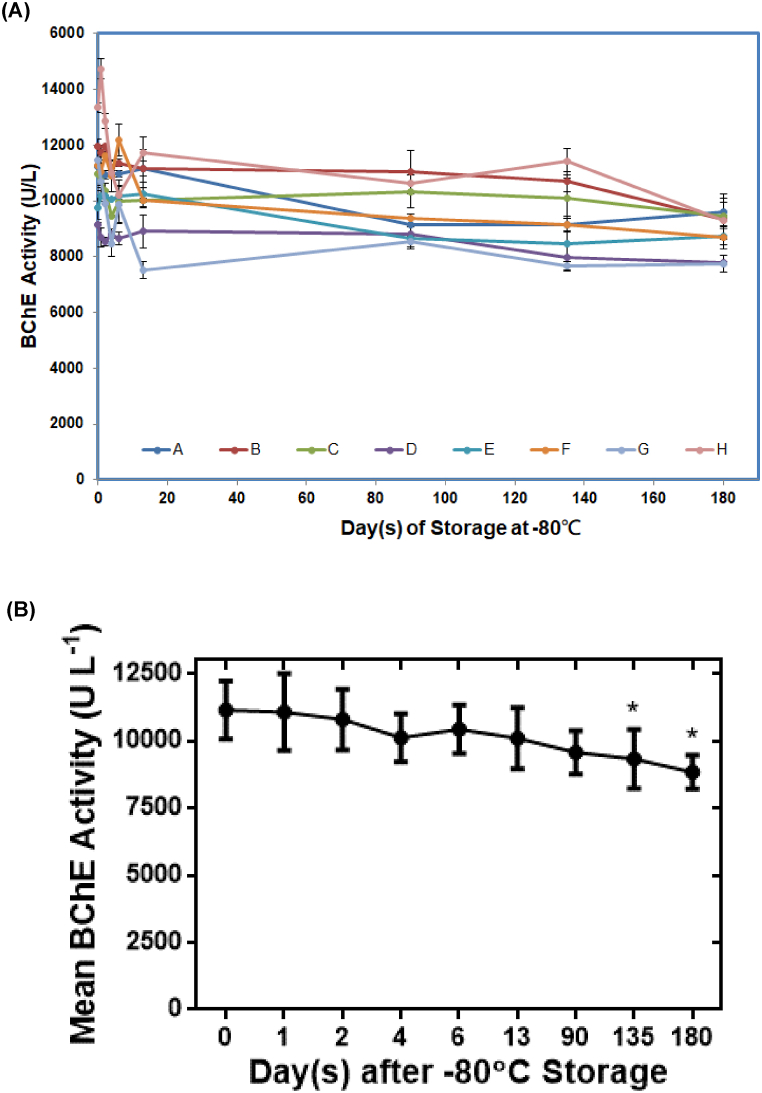
Changes of serum BChE activity and correlation of mean serum BChE activity during storage at −80 °C over a period of 180 days. (A) Serum samples were obtained from eight volunteers consisting of four males (Volunteers A, C, E and H) and four females (Volunteers B, D, F and G) and were determined BChE activity according to the sensitive fluorescence turn-on assay [14] after a specified storage time at −80 °C. Each serum sample was measured at least three times in order to calculate the averaged BChE activity and the standard deviation (the error bar). (B) Values of mean serum BChE activity were derived from eight volunteers' averaged BChE activity analyzed on the specified time of storage at −80 °C. Each mean serum BChE activity was calculated according to Marginal Means of repeated measures ANOVA with a 95% confidence interval (the error bar). *p-value < 0.05 compared with the values obtained on Day zero.

Eight volunteers, specifically four males and four females, were recruited to donate the blood used in this study. Fresh serum and the corresponding serum aliquots stored in the −80 °C freezer for 1, 2, 4, 6, 13, 90, 135, or 180 days were analyzed by our recently developed fluorescence turn-on assay [14] and determined BChE activity (Fig. 1A). All but two (Volunteer H at Days 0 and 1) measurements provided averaged serum BChE activity within the normal physiological range of BChE activity, 5900–13,200 U L^−1^ [15]. In addition, results from independent t-tests showed that there was no significant difference in values of gender-mean BChE activity in serum from male and female volunteers (analysis not shown). Therefore, simple traces as shown in Fig. 1A of averaged BChE activity versus −80 °C storage time in days were insufficient to establish valid criteria of preanalytical conditions for sample storage in order to unambiguously reproduce measurements of BChE activity after prolonged storage.

### 3.2 Statistical analysis to validate the effect of −80 °C storage time on serum BChE activity

The time-dependent BChE activity values of the serum stored at −80 °C were further analyzed by repeated measures ANOVA, which demonstrated that the serum stored at −80 °C for 135 and 180 days yielded the mean BChE activity values significantly diverse from the rest of the BChE activity values (*F* = 9.415, *p* < 0.001; Table 1). The results were also replotted as Fig. 1B. Advanced studies of Sidak multiple comparisons test using the mean values of averaged BChE activity at a specific −80 °C storage time corroborated our finding that the mean BChE activity values from the samples stored for 135 and 180 days were statistically different from that of fresh serum (Day 0) (Table 1). Serum stored at −80 °C for 90 days, therefore, represents a maximum limit able to reliably reproduce values of BChE activity in fresh serum samples.

**Table 1:** Statistical analysis of mean values of BChE activity by Sidak multiple comparisons test.

Storage Time at −80 °C	Means of BChE activity	Sidak multiple comparisons test	
	M ± SD	Mean difference from Day 0	*p*-value
Day 0	11146.5 ± 1285.39	–	–
Day 1	11068.9 ± 1717.58	77.6	1.000
Day 2	10792.3 ± 1355.45	354.2	.997
Day 4	10112.3 ± 1074.34	1034.3	.738
Day 6	10423.6 ± 1074.87	723.0	.996
Day 13	10093.9 ± 1363.96	1052.6	.899
Day 90	9567.8 ± 963.34	1578.7	.082
Day 135	9326.6 ± 1319.68	1819.9	.027
Day 180	8839.3 ± 739.67	2307.2	.023

## 4 Discussion

The current study determined sources of preanalytical variability affecting reproducible measurements of BChE activity in serum. We were particularly interested in understanding the changes in serum BChE activity stored at −80 °C for a period of 180 days. The serum storage temperature at −80 °C was chosen because −80 °C freezers are typically available in clinical settings capable of performing biochemical analysis on patient samples. We decided to study variations of serum BChE activity during a 180-day storage at −80 °C since that was proposed to be long enough to reveal significant decay of BChE activity (Fig. 1A). Indeed, our statistical analysis of the BChE activity results by repeated measures ANOVA and Sidak multiple comparisons test demonstrated that 90-day storage at −80 °C was the maximum allowed time in order to acquire serum BChE activity equivalent to that of fresh serum (Fig. 1B and Table 1).

The conclusion that serum BChE activity is stable and unchanged only up to 90-day storage at −80 °C contradicts the results of a previous study [12], which suggested that serum BChE activity experienced no change during storage at −20 °C for one year. Previous research, however, relied on the insensitive colorimetric Ellman assay [16] to quantify BChE activity in serum and, thus, might be incapable of sensitively detecting changes in BChE activity. In contrast, we employed a sensitive fluorescence turn-on assay [14] to measure serum BChE activity throughout this study. The fluorescence turn-on assay is characterized by a low limit of detection and a broad dynamic detection range [14] and is naturally more appropriate to accurately determine serum BChE activity and to identify the critical duration time essential to preserving serum BChE activity while stored at −80 °C.

Regarding the sample size, this study measured BChE activity in the serum from only eight volunteers because eight serum samples are the maximum that we can analyze by the fluorescence assay in a fluorescence spectrophotometer in a single day. We are developing high-throughput fluorescence assays for quantification of BChE activity in samples, which will expand our capacity to analyze a greater number of serum samples in one day and will further corroborate and scrutinize the results provided in the study.

This study also demonstrated that statistical analysis of the BChE activity data was essential for establishing that BChE activity could be reproducibly measured in serum samples stored at −80 °C for up to 90 days (Fig. 1B and Table 1). Similar statistical analysis approaches will be exploited on studies of BChE activity data acquired from high-throughput assays in the future.

In summary, we have provided crucial information that BChE activity in fresh serum could be reproduced using the same serum samples when stored at −80 °C for up to 90 days. This knowledge concerning the stability of BChE activity during storage will assist clinicians in properly handling serum samples, determining meaningful BChE activity, and provide pertinent treatments to patients.

## Author Contributions

Chien-Hui Huang, Validation, Formal analysis, Investigation; Yi-Ting Chang, Validation, Formal analysis, Writing - Original Draft; Scott Severance, Methodology, Writing - Original Draft, Writing - Review & Editing, Visualization; Jui-Ying Feng, Methodology, Writing - Original Draft, Writing - Review & Editing, Visualization; Sin-Yu Hou, Formal analysis, Investigation; Ming-Mao Gong, Formal analysis, Investigation; Chi-Ching Hwang, Methodology, Writing - Original Draft, Writing - Review & Editing; Chia‐Yen Dai, Conceptualization, Methodology, Resources; Jeh-Jeng Wang, Resources; Tzu-Pin Wang, Conceptualization, Methodology, Formal analysis, Data Curation, Writing - Original Draft, Writing - Review & Editing, Visualization, Supervision, Project administration, Funding acquisition.

## Declaration of competing interest

The authors declare that they have no known competing financial interests or personal relationships that could have appeared to influence the work reported in this paper.
